# Using the PEBIC criteria to assess the quality of responses provided during a Family Planning radio program

**DOI:** 10.3389/fgwh.2025.1499341

**Published:** 2025-03-11

**Authors:** Babafunke Fagbemi, Abiodun Adegbenro, Toyin Akande, Charles Udennaka, Adaora Uzoh-Ntiwunka, Bukola Toriola, Adenike Ayodele

**Affiliations:** Research Monitoring Evaluation and Learning Department, Centre for Communication and Social Impact (CCSI), Abuja, Nigeria

**Keywords:** Hormonal IUDs, contraception, radio programming, SBCC, PEBIC criteria, Family Planning (FP)

## Abstract

**Introduction:**

Radio remains a dominant source of information, in Sub-Saharan Africa, with 75% of the population relying on it. In Nigeria, radio reaches over 70% of citizens and stands as a vital tool for behavior change. Tailoring radio programs to address needs and cultural contexts of communities has proved essential for building trust and influencing behavioral change. Traditional evaluation of radio interactive programs focus on listener engagement metrics but overlooks the evaluation of the quality of the resource person's responses to the caller's questions. This study aimed to assess the quality of information delivered by resource persons during the interactive segments of a Family Planning (FP) radio program using a criteria.

**Methods:**

A qualitative analysis was conducted on caller questions and resource person responses from FP radio programs in three Nigerian states. A customized ranking system based on “Presentation,” “Empathy,” “Provider's Bias elimination,” “Information correctness,” and “Context Specificity” (PEBIC) was used by FP experts to assess the resource person's response quality. The scores for the responses were segmented and categorized into low, moderate and high quality using the mean, and standard deviation then analyzed using SPSS version 20.

**Results:**

For the technical segment of the radio program, while individually assessing each item on the criteria, the quality of responses was high, with an average of 91.2%. For the entertainment education segment of the radio program, while individually assessing each item on the criteria, the quality of responses with empathy was low (28%), however, the quality of responses for other criteria was high, with an average of 79.8%. Furthermore, in the overall quality of response for the technical segment, the responses given by the resource persons were of high quality (81%) while those of the entertainment education segment were of moderate quality (58%).

**Conclusion:**

The radio program excelled in delivering technical information but fell short in fostering adequate empathy during the entertainment education segment. Additional training for resource persons to hone their skills in embedding their message delivery in an empathetic frame thus strengthening the emotive connection with the audience is key. This could significantly improve the program's overall impact.

## Introduction

Radio, emerging in the late 19th century, evolved from telegraphy into a global broadcasting tool, with Marconi's 1901 transmission and Fessenden's 1906 broadcast marking key milestones (1, 2). Despite the advancements in digital technology and telecommunication, radio remains highly influential, particularly in Sub-Saharan Africa, where 75% of the population relies on it as their primary source of information (3, 4). Nigeria serves as a prime example, where radio programming holds immense potential, reaching over 70% of the population and serving as a powerful tool for promoting health-related behavioral change across diverse demographics (5, 6).

The cost-effectiveness, extensive reach, and ability to penetrate remote regions with limited internet connectivity make radio indispensable for driving societal transformations, including influencing health-related behaviors (7). Moreover, its capability to transcend geographical and linguistic barriers allows radio broadcasts to effectively engage audiences across Nigeria's 36 states and 774 local government areas providing a distinct advantage, particularly in rural areas with limited internet access (8).

Radio allows for a more intimate relationship with listeners than other media since the human voice in familiar languages can elicit emotions, develop trust, and drive action (9). Listeners often cultivate parasocial relationships with radio personalities, enhancing their receptivity to messages and calls to action (10, 11). This phenomenon can be particularly potent in delivering health messages, as recent research indicates that audio narratives, including radio dramas, are effective tools for promoting positive health behaviors, such as those associated with Family Planning (FP) (12, 13). Family Planning radio programs play a crucial role in disseminating information and promoting awareness about reproductive health and FP choices (14). These programs cater to diverse audiences, often in remote areas with limited access to other information sources. Tailoring radio programming to meet the individual needs and cultural idiosyncrasies of Nigerian communities builds trust and increases the possibility of engaging with listeners, favorably affecting their behavior (12).

## The use of resource persons on information dissemination on radio program

Traditionally, assessments of radio programs including those focused on FP have primarily concentrated on audience engagement metrics like listenership and call-in rates. While these metrics offer valuable insights, they do not directly evaluate the quality of the resource person's responses to the caller's questions (15).

Resource persons are essential in radio programs that have interactive segments, specifically programs with opportunities for listeners to ask questions in real time through calls or text messages (16). This gives room for information dissemination, providing expertise, credibility, and diverse perspectives that enhance content quality and impact. Experts such as academics, researchers, and professionals offer specialized knowledge and engage listeners with engaging delivery, ensuring accuracy and comprehension (17). Their credibility fosters trust and encourages audience engagement, particularly when addressing complex or controversial topics. Including resource persons from various backgrounds enriches discussions, offering multiple viewpoints and insights into real-world experiences (18). Effective utilization involves careful selection based on relevant expertise and strong communication skills, thorough preparation, and facilitation of engaging dialogue, as exemplified in a public health radio program addressing sanitation and hygiene practices with contributions from a medical doctor, sanitation engineer, and community leader (19).

This paper proposes a novel approach to assessing FP radio programs by focusing on the quality of responses from the resource person. By shifting the focus from audience response to the program's content and quality of delivery, evaluating the resource person's responses is of paramount importance for several reasons. This unique approach offers valuable insights by identifying areas for program improvement, highlighting where the resource person can enhance their responses to better engage and inform the audience. Moreover, it contributes to the dissemination of accurate and reliable information on FP through the program, ensuring quality information reaches the audience. Additionally, by encouraging the development of communication skills specific to FP radio shows, this approach promotes more engaging and impactful programs.

## Get it Right radio program

The “Get it Right” radio magazine program was a 30-minute live program that included an interactive phone-in segment. The program was broadcast in English, Pidgin, Yoruba, and Hausa, which are the predominant languages of the project states. The program's format was kept simple to emphasize the content and the phone-in segment. Additionally, radio jingles that promote FP and amplify the testimonials of a male whose partner uses FP were played. These radio jingles were aired at least three times a week and twice during the radio program.

The magazine program was divided into two segments and contained 20 episodes in total. It combined basic information on FP with lifestyle approaches. The first eleven episodes (segment one- technical) focused on technical FP topics, while the last 9 episodes (segment two- entertainment education) were designed using an entertainment education format that chronicled the journey of a Hormonal Intra-Uterine Device (H-IUD) user.

Segment one covered modern FP and Childbirth Spacing (CBS) methods in Nigeria, exploring the benefits of FP, who can use it, and the importance of communication between couples. It also dived into specific methods like the H-IUD, addressing myths and misconceptions surrounding it, the role of religion, culture, healthcare workers, and the overall impact of FP/CBS on health.

Segment two outlined a more persuasive strategy to promote H-IUD as a FP method infusing the story of a satisfies HIUD user into the contents of the radio program to inform potential users and listeners about the benefits and accessibility of H-IUDs.

Six radio stations were selected to air the “Get it Right” radio program from the 17th of July to the 15th of December 2023, across the project states. In Delta state, Trend FM and Crown FM were chosen, while Parrot FM, Splash FM, and Gravity FM were selected for Oyo state, and Plateau Radio and Television corporation (PRTV) Peace FM was included for Plateau state. The selection was based on considerations of regional reach, diverse audience demographics, and the need to accommodate language and cultural sensitivities. Each station was chosen to explore urban-rural distinctions, religious impacts, and unique media environments within their respective states. This meticulous approach allowed for targeted interventions to address distinct FP challenges and dispel prevalent misconceptions. A team comprising experienced On-Air-Personalities (OAPs) and Resource persons were identified across the states to deliver the content of the episodes. The OAPs were selected based on their extensive experience in FP programs, while the resource persons are State FP Coordinators who have been trained and have relevant experience in the subject matter.

## Objective of study

To assess the quality of responses provided by resource persons to callers' questions during the radio program.

## Methodology

### Study design

The assessment was a cross-sectional study which utilized a qualitative approach to gather and assess questions asked by callers and responses given by resource persons during an FP radio program.

### Study area

The assessment was done on calls and responses from the “upgrade your life” FP radio programs on stations in Delta, Oyo, and Plateau states.

Sample size estimation:

The estimated sample size for the study was calculated as:

$$n=\frac{{\left(Z_{1-\alpha /2}\right)}^{2}P(1-P)}{d^{2}}$$

*n* is the sample size

• *Z*_1−*α*/2_ is standard normal varied alpha level (5%) = 1.96
• *P* is the expected proportion of radio listenership in Nigeria = 77% (20).
• *d* is precision set at 5% (0.05)
• 10% non-response rate
  $$n=\frac{{\left(Z_{1-\alpha /2}\right)}^{2}P(1-P)}{d^{2}}$$
  $$n=\frac{{\left(1.96\right)}^{2}\times \;0.77(1-0.77)\;}{{\left(0.05\right)}^{2}}$$
  $$n=\frac{3.84\times 0.77\times 0.23}{0.0025}=272$$
  $$\begin{array}{rl} \mathrm{Calculating}\;\mathrm{the}\;10\text{\%}\;\mathrm{non}\text{-}\mathrm{response} & =10\text{\%}\;\mathrm{of}\;272\\ & =10/100\times 272\\ & =27 \end{array}$$

The calculated sample size needed for the study = 299.

### Data collection/extraction

Qualitative data was gathered through the transcript from the audio recordings of calls from callers on the FP radio program. A total of 723 callers' questions and responses from the resource persons were identified throughout the life span of the radio program (for a period of six months). All were purposively included in the study.

### Resource persons response ranking

A customized ranking sheet (checklist) was developed to rate the resource person's responses to questions asked by the callers. This was developed based on a set of criteria to guide the rating of the responses given and later validated with evidence derived from literature.

These criteria are “**P**resentation”, “**E**mpathy”, “provider's **B**ias elimination”, “**I**nformation correctness”, and “**C**ontext Specificity”. Each criterion was categorized as good or bad through a “0” or “1” point. Zero stands for bad and one stands for good. If a response had all the criteria correct, it would be assigned a total of “5 points” or scored according to the set of criteria that was marked correct.

Family Planning experts read through each response using the World Health Organization (WHO) Frequently Asked Questions (FAQ) “Your Contraception guide” (21, 22) and their prior experience of over ten (10) years in FP programming to ascertain if the resource persons conveyed the right information using the criteria developed that is: presenting their thoughts well and demonstrating empathy. The Family Planning experts also ascertained if there were any elements of provider bias in the response and whether the contents of the responses provided were specific to the context of the questions asked. The items on the criteria are defined below:

**Presentation:**
This refers to the way the resource person delivers their response. As outlined by Ekholm et al. (23), the answer should be clear, and concise, and avoid irrelevant details. It should demonstrate a strong understanding of the subject matter and be well-organized with a logical flow of thought.
**Empathy:**
This criterion focuses on the resource person's ability to connect with the caller on an emotional level. As suggested by Houtepen et al. (24), it involves understanding the caller's emotions and responding with sympathy and respect. The resource person should use language appropriate to the caller's level of understanding.
**Bias Elimination:**
This ensures responses are objective and avoids promoting personal opinions or the provider's own biases. Kreuter et al. (25) emphasize the importance of distinguishing personal views from information. The resource person should present a balanced perspective to allow the audience to form their own conclusions.
**Information Correctness:**
This criterion ensures the accuracy of the information provided. The Family Planning experts verified the information against the WHO FP FAQs and ensured responses avoided subjective statements, unconfirmed information, or irrelevant tangents (21).
**Context Specificity:**
This assesses if the resource person directly addresses the caller's specific question. As highlighted by Myers et al. (26), generic responses or information not relevant to the topic should be avoided. The resource person should demonstrate expertise in the specific context area and provide insights that directly connect to the caller's query and context.

### Data analysis

Family Planning experts were engaged to rate the quality of resource persons' responses to questions asked during the radio program based on the PEBIC criteria. The engagement and quality of the responses on the radio program were assessed also bearing the standards stipulated in the radio program design documents, communication strategy and the “your contraception guide” on frequently asked questions and definitions of FP practices in mind. This analysis generated a score for each of the items on the criteria and the ranked scores were analyzed using SPSS version 20.

### Ranked scores summation and categorization

Using the Mean (M) and Standard Deviation (SD) of the summed scores for each response. Resource person's responses with scores less than or equal to the Mean—SD were classified as “Low quality”, scores between the Mean—SD and Mean + SD were classified as “Moderate quality” and responses with scores greater or equal to the M + SD were classified as “High quality”.

### Quality assurance

Each episode of the radio program was recorded. A full transcript of the 30 min radio program was done of the questions asked by callers and the resource person's responses were extracted. A procedure for applying the PEBIC criteria was deployed and strictly followed. The criteria for selecting the FP experts that reviewed the responses of the resource persons was developed. After an FP expert reviews the resource person's responses, the same responses are given to a different FP expert to validate, and the reviews are compared to ensure accuracy. A checklist that consists of the PEBIC criteria was also developed to ensure seamless ranking.

## Results

### Radio listeners’ engagement with the Get it Right radio program

#### Distribution of questions from callers

**Figure 1:**
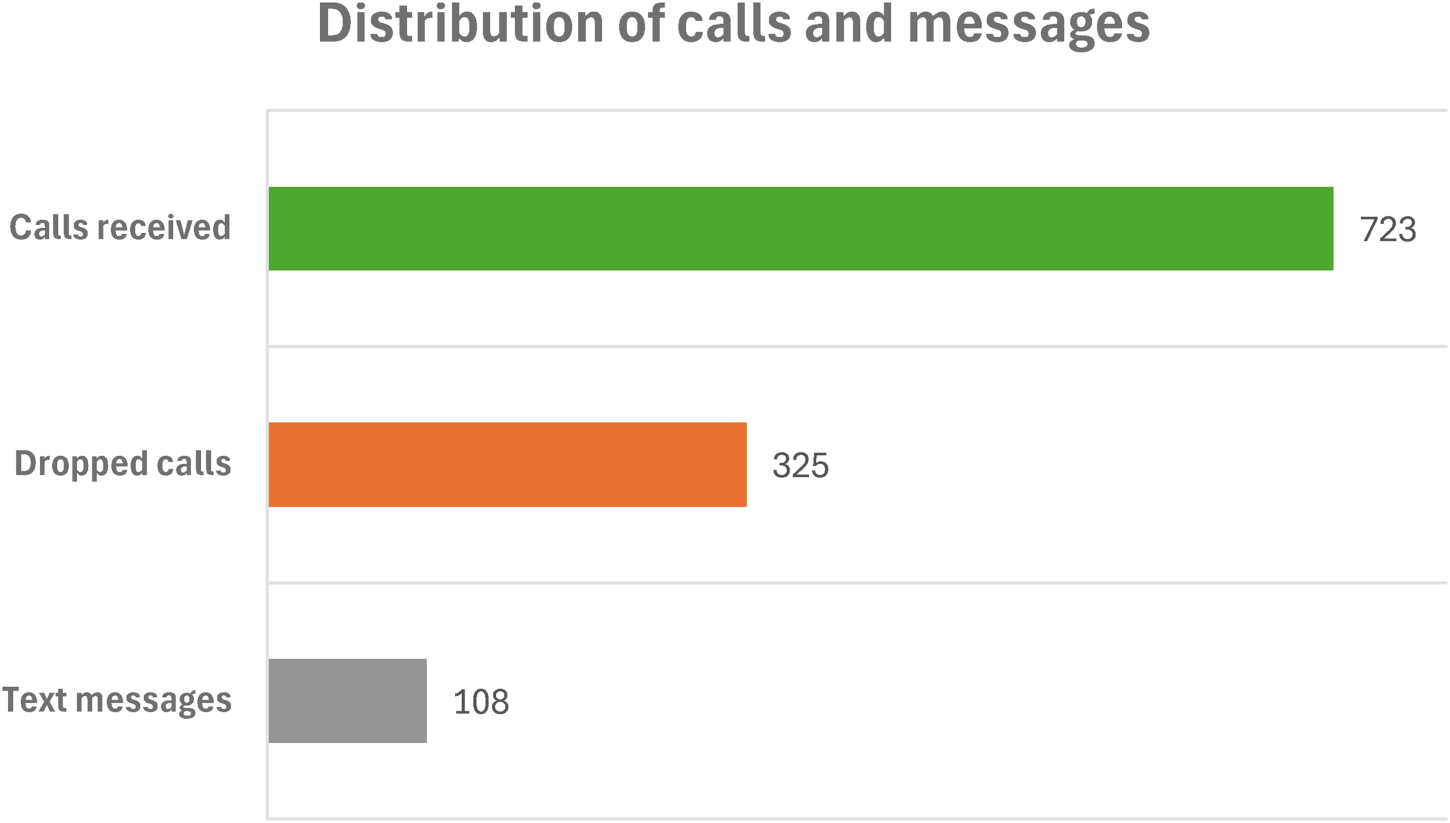
Distribution of calls and messages across the radio stations.

The “Get it Right” radio program had a lot of engagement from the listeners. The radio program recorded 1,048 call attempts across the radio stations of which 723 calls were received while 325 calls were either dropped or missed due to high call traffic and poor network reception respectively. The Figure 1 below shows the distribution of calls and messages received during the radio program.

### Quality of the resource persons' responses

The quality of the responses delivered by the resource persons during the project's radio program was tested by ranking the responses based on the PEBIC criteria. The responses were ranked using “0” for an incorrect response (No) and “1” for a correct response (Yes). The quality of the response was assessed in two ways: (a) Assessing each of the items that form the criteria individually and (b) Assessing all the criteria together (overall).

**Figure 2:**
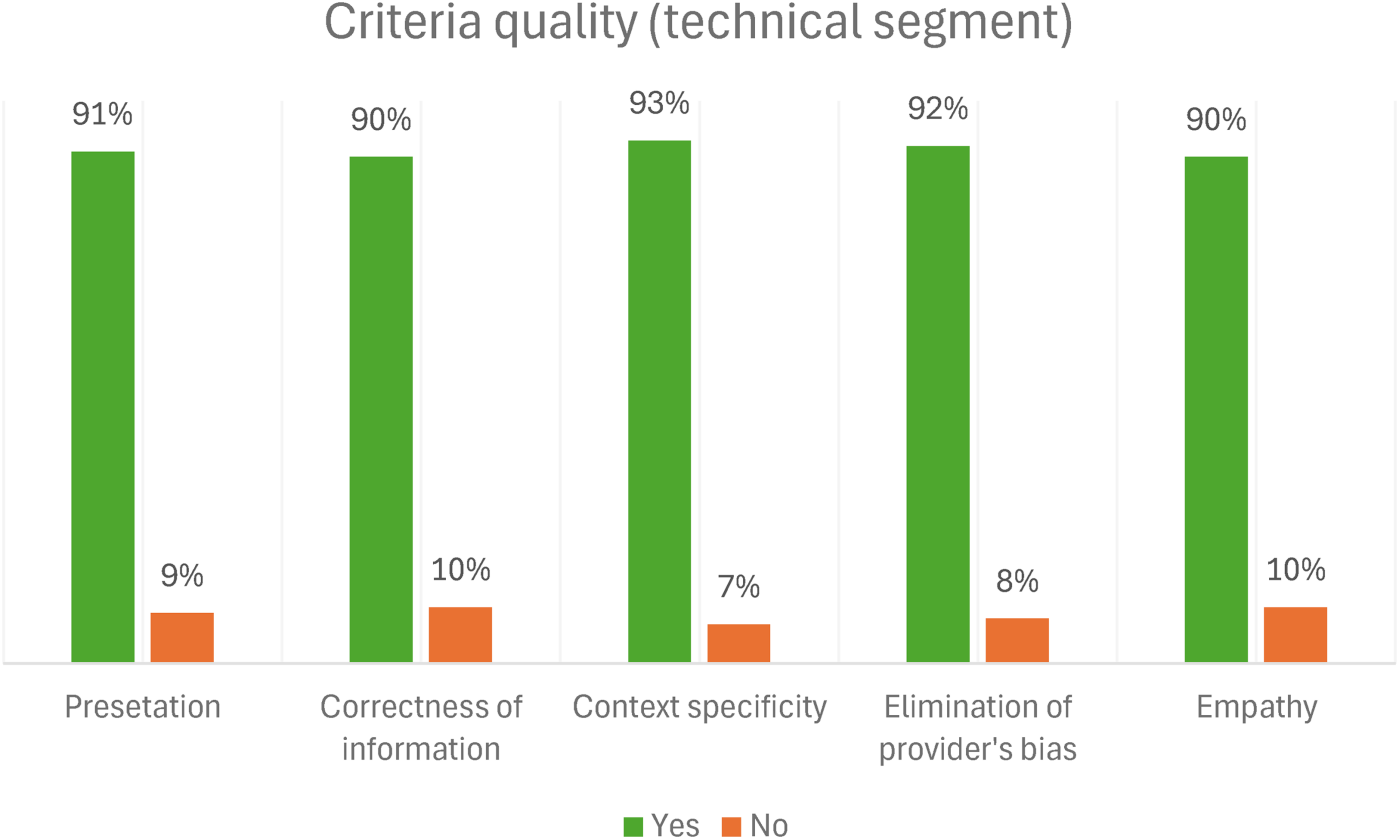
The quality of resource persons' responses to callers' questions in the technical segment.

**Figure 3:**
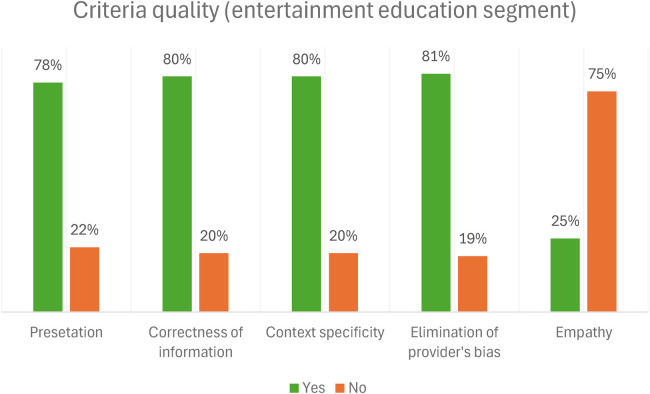
The quality of resource persons' responses to callers' questions in the entertainment education segment.

When assessing each item individually, for the technical segment of the radio program, the quality of responses for the criteria was high, with an average of 91.2%. When assessing each item individually for the entertainment education segment, the quality of responses with empathy was low (25%). However, the quality of responses for other criteria was high, with an average of 79.8%. Figures 2, 3 below show the criteria for the technical and entertainment education segment and their associated percentages of the radio program.

When assessing all the criteria together, the overall quality of the responses for the technical and entertainment education segments was derived by summing all the scores for the responses of the individual items (on the criteria) in each segment and categorizing them into low, moderate, and high quality using the mean and standard deviation.

**Figure 4:**
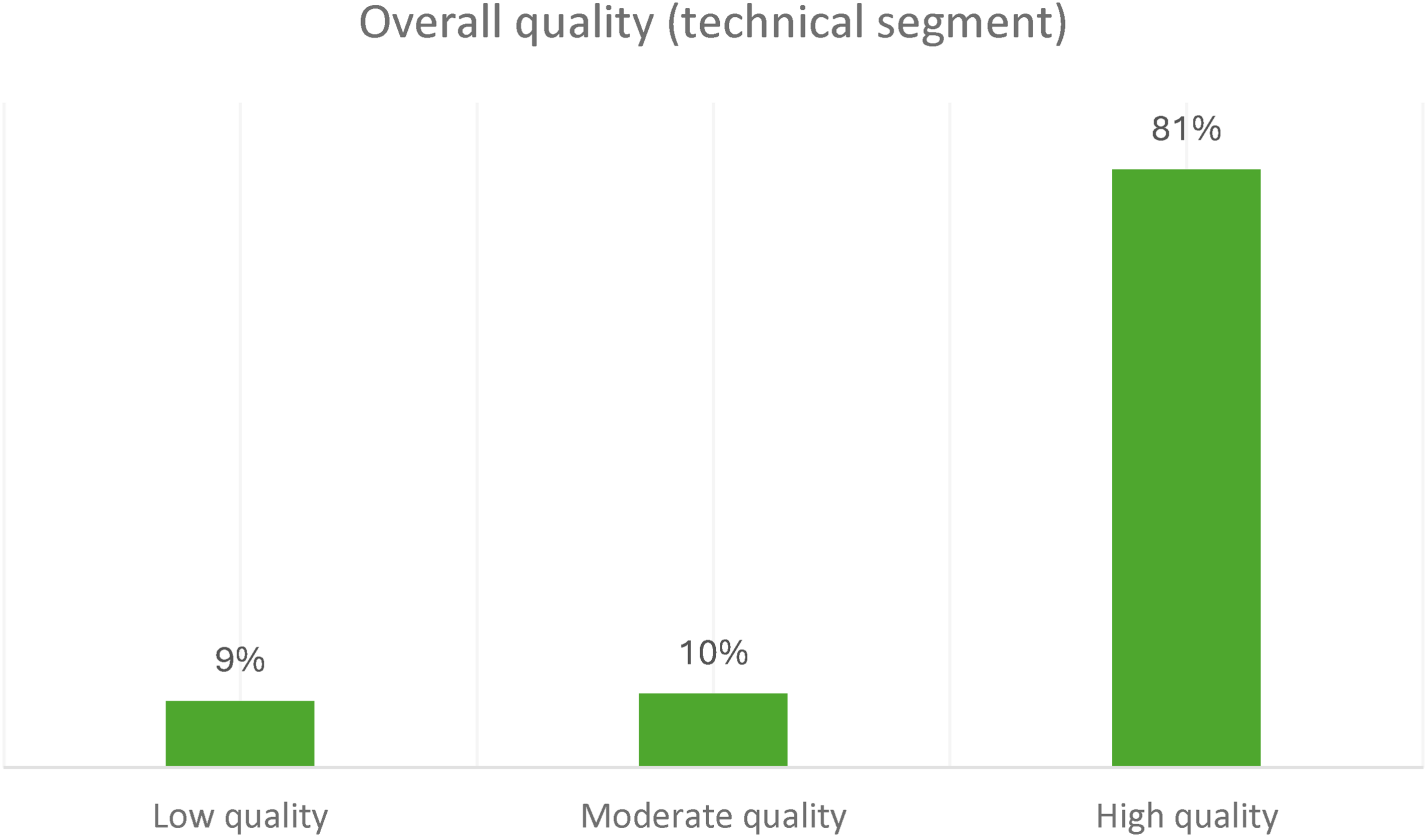
The level of quality of the overall response for technical segment.

For the overall technical segment, the results show that the responses given by the Resource persons were of high quality. Figure 4 below shows the level of quality of the overall response for the technical segment.

**Figure 5:**
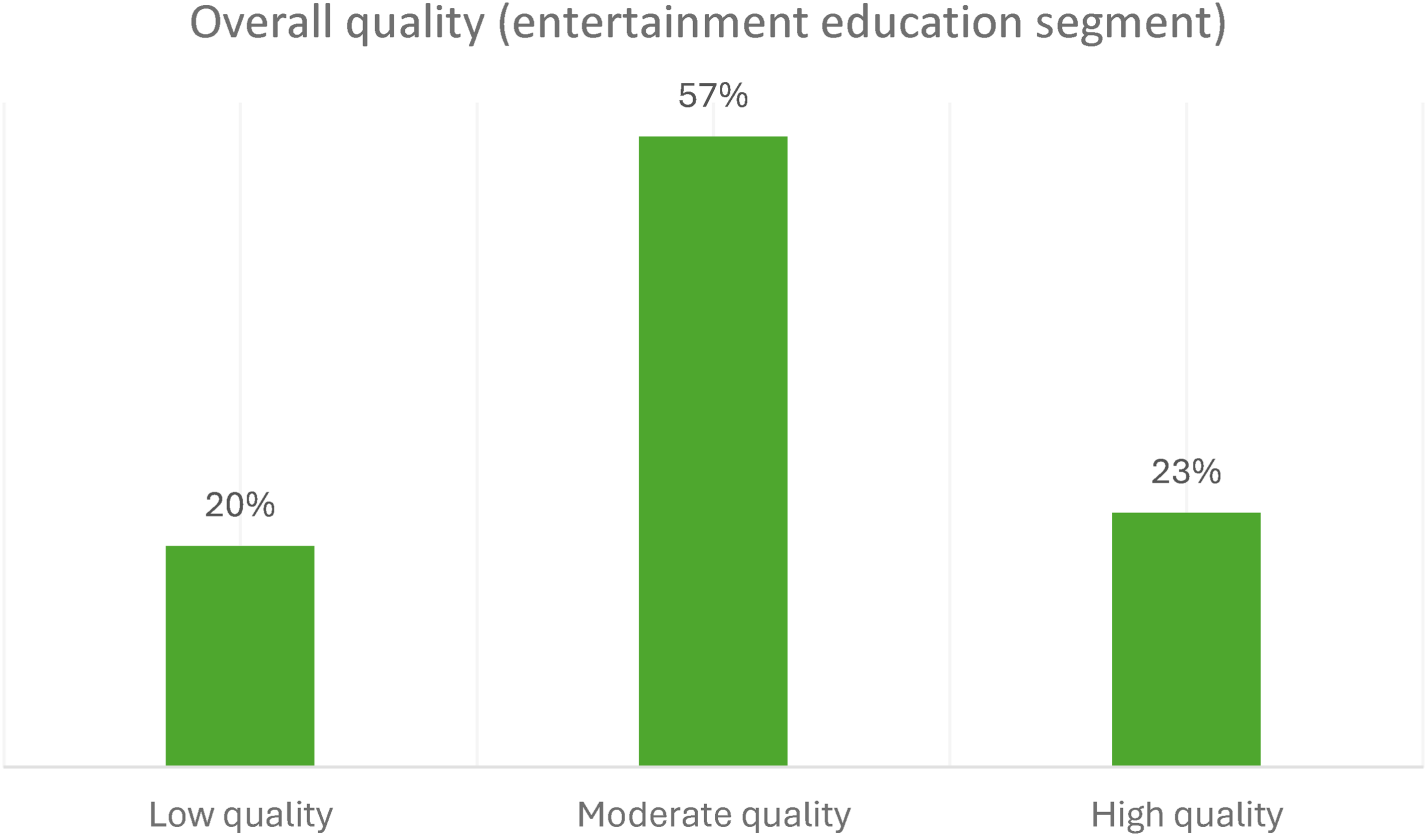
The level of quality of the overall response for the entertainment education segment.

For the entertainment education segment, the results showed that most of the responses given by the resource persons were of moderate quality (57%). Figure 5 below shows the level of quality of the overall response for entertainment education.

## Discussion

The assessment of resource persons' responses to caller questions utilized a comprehensive ranking using the PEBIC criteria to provide valuable insights into the effectiveness of the Get it right radio program. In the technical segment, resource persons exhibited high responses with effective delivery of clear and accurate technical information which aligns with best practices in evaluating health communication interventions highlighted by (27), affirming the Get it right radio program's efficacy in equipping participants with the requisite knowledge and skills to communicate factual details in a manner that connects with the audience.

Different studies have utilized various criteria to assess resource person responses to caller questions. Some studies emphasize technical aspects such as response accuracy and completeness (23), while others prioritize caller-centered communication skills such as active listening and open-ended questioning (28). Myers et al. (26) delved into explaining how cultural competency is in ensuring information resonates with diverse audiences.

Although Resource persons demonstrated proficiency in aspects such as presentation style, context specificity, information correctness, and bias elimination, their capacity to cultivate empathy with the audience was noticeably lacking. Nurturing empathy during communication, as underscored by Houtepen et al. (24), emphasizes the critical role of empathy in health communication for fostering trust and catalyzing behavior change. Empathy serves as a bridge that connects individuals emotionally, fostering a deeper understanding and resonance with the message being conveyed (29). In the context of health communication, empathetic engagement can empower individuals to embrace positive health behaviors and make informed decisions regarding their well-being (30). Thus, enhancing empathy in resource persons' interactions can significantly amplify the impact of health communication initiatives, ultimately leading to improved health outcomes and societal well-being (31).

Our assessment was a holistic evaluation approach, which considers both technical proficiency and audience engagement. While the accurate delivery of information remains paramount, neglecting the emotive connection fostered by empathy could impede behavior change. By integrating empathy alongside established criteria, this study offers a more comprehensive assessment of Resource person effectiveness, particularly within the entertainment education format.

Fortunately, several techniques can bridge the empathy gap and enhance the effectiveness of entertainment education segments. Mohseni-Moghadam et al. (32) advocate for incorporating storytelling, active listening, and acknowledging listener concerns as fundamental strategies for fostering adequate empathy in health communication. Future research could explore the specific impact of these techniques on empathy scores within entertainment education settings.

By addressing the identified empathy gap and conducting further research, we can refine capacity building formats to equip resource persons with a comprehensive skill set for effective health communication, with an emphasis on delivering technical information in an empathetic manner, ultimately facilitating positive behavior change within the target audience.

## Conclusion

The implemented multi-criterion ranking system proved effective in evaluating the resource persons' response quality. This assessment evaluated the quality of a radio program resource persons' responses using the PEBIC criteria (Presentation, Empathy, Bias elimination, Information correctness, Context Specificity) across two segments: technical and entertainment education.

The findings demonstrate a difference in the quality of responses between the segments based on empathy. The technical segment achieved a high average score across all PEBIC criteria, indicating clear, unbiased, and accurate presentations. However, the entertainment education segment, while maintaining high quality in other criteria, showed a weakness in empathy.

Furthermore, the overall quality assessment confirmed this disparity. The technical segment achieved a high-quality rating, whereas the entertainment education segment fell within the moderate quality range.

In conclusion, the radio program excelled in delivering technical information but fell short in promoting sufficient empathy during the entertainment education segment. Additional capacity building for resource persons on incorporating audience connection and emotional understanding within the entertainment format could significantly improve interactive radio program's overall impact.

## Data Availability

The original contributions presented in the study are included in the article/Supplementary Material, further inquiries can be directed to the corresponding author.
